# Soil meso- and micro-fauna community in response to bamboo-fungus agroforestry management

**DOI:** 10.1038/s41598-022-20738-y

**Published:** 2022-09-30

**Authors:** Jiancheng Zhao, Miao Liu, Jun Xu, Zhenya Yang, Qin Li, Chunju Cai

**Affiliations:** 1 Zhejiang Academy of Forestry, Zhejiang Provincial Key Laboratory of Bamboo Research, Northwest Zhejiang Bamboo Forest Ecological Station, Hangzhou, 310023 Zhejiang China; 2 International Center for Bamboo and Rattan, Key Laboratory of Bamboo and Rattan, Beijing, 100102 China; 3 Natural Resources and Planning Bureau of Deqing County, Deqing, 313200 Zhejiang China

**Keywords:** Ecosystem ecology, Forest ecology, Forestry

## Abstract

Bamboo-fungus agroforestry management is an ecological model of sustainable production of moso bamboo forest, and *Stropharia rugosoannulata* has been widely planted in moso bamboo forest. However, little attention has been paid to soil fauna community in bamboo-fungus agroforestry system. Thus, the aim of this study was to investigate the response of soil fauna communities to agroforestry management, and to explore the relationships between soil fauna communities and soil properties. An experiment with 0, 1, 2 and 3 years of planting was carried out in an existing moso bamboo forest. The community composition of soil meso- and micro-fauna was investigated, and the soil properties were determined. Results showed that a total of 2968 individuals of soil meso- and micro-fauna, belonging to 8 classes and 13 groups were detected. The group number and density of soil fauna was highest right and then decreased. Planting *Stropharia rugosoannulata* in moso bamboo forest increased the density of dominant groups, but did not change its composition. Shannon-Weiner diversity index (H), Margalef richness index (D) and Density-Group diversity index (DG) were the highest one year after planting the fungus, while Simpson dominance index (C) was the lowest in the meantime. Contents of soil moisture (SMC), organic matter (SOM), total nitrogen (TN), total phosphorus (TP) and total potassium (TK) increased first and then decreased with the increase of planting years, peaking at 1 year after planting, while the pH value continued to increase. Responses of soil fauna community were associated with soil physicochemical properties. Redundancy analysis (RDA) showed that SOM was the main environmental factor driving the variation of soil fauna community, followed by TP and TN. In conclusion, planting *Stropharia rugosoannulata* in moso bamboo increased the diversity and abundance of soil fauna communities due to its contribution to abundance of organic matter and supply of nutrients.

Moso bamboo (*Phyllostachys edulis* (Carrière) J. Houzeau) is one of the most important forest resources in Southern China, which is characterized by its fast growth during sprouting and rapid biomass accumulation^1–3^. As a major non-wood resource, moso bamboo forest plays an important role in socioeconomic and international trade^3,4^. The yields of forest products (bamboo timber and bamboo shoots) have been stagnant at the current level of input^2^. Furthermore, the phenomenon of abandonment caused by the decrease of the bamboo shoot price and timber price and the increase of the labor costs has received an increasing attention in recent years, which reduced the enthusiasm of bamboo farmers for management^5,6^.

Agroforestry management determines the efficiency of forest land use, spatial utilization rate, diversity of forest products, farm benefit, and ecological functions^7,8^. Interplanting of edible fungus in moso bamboo forest is one of the systems of agroforestry, and bamboo-fungus agroforestry management is an ecological model of sustainable production of moso bamboo forest^9^. *Stropharia rugosoannulata* is one of such mushrooms that is recommended by United Nations Food and Agriculture Organization (FAO) for cultivation in developing countries^10,11^. It has high nutritional value for human and certain pharmacological functions^12^. Additionally, it lives on decomposing grasses and the cultivation technique is simple. Therefore, this mushroom has a long history of cultivation in moso bamboo forest in China.

Soil fauna plays many important roles in soil ecosystem, such as litter decomposition, nutrient cycling, maintenance of soil structure and stability, and improvement of soil physicochemical properties^13–17^. As a result, soil fauna is an excellent indicator of soil quality^18^. Previous studies showed that soil fauna was sensitive to environmental changes^18,19^, and it was applied to indicate certain features of soil fertility. When soil environmental changes such as moisture exceeded limitation of the body adaption and regulation, the survival and reproduction of soil fauna could be affected^20^. Moreover, long-term fertilization in cropland affected soil properties by changing the species and quantity of plant residues and root exudates, which subsequently changed the diversity and composition of soil fauna communities by changing the ecosystem of the soil fauna^21^. Soil fauna first fracture the residues, thereby increasing the surface area available to microbes and through residues decomposition by microbes the availability of nutrients increases^22^. The quantities of groups and individuals of soil fauna communities in the fertilization regimes with crop residues returned were much greater than in the other sampling times, and a significant correlation between the main soil properties and the indices of soil fauna indicators was found^23^.

Previous studies showed that intercropping in *Ginkgo biloba* forest increased the biodiversity of soil fauna^24^, and returning of organic matter in rice paddy increased the populations, groups and diversity of soil fauna^25^. These results indicated that the abundance and diversity of soil fauna community were significantly influenced by different tillage managements. During the cultivation of *Stropharia rugosoannulata*, organic matters (rice chaff and straw) were introduced into moso bamboo forest, which affected the soil microenvironment and soil properties^26^. Research on the mushroom yield, suitable bamboo forest density, soil physicochemical properties and microbial characteristics in the bamboo-fungus agroforestry system has received wide attention nowadays^26^. The extra economic return from the fungus was increased, and the diameter at breast height (DBH, 1.3 m) of new bamboo was also improved^26^. However, it is still unknown whether the system is beneficial to soil fauna and thereby contributes to improve soil quality.

Therefore, the purposes of this study were to investigate the diversity and abundance of soil meso- and micro-fauna following different years of mushroom growing in a bamboo-fungus agroforestry system, and to determine the relationship between major soil fauna groups and soil properties.

## Results

### Soil fauna community composition

**Figure 1:**
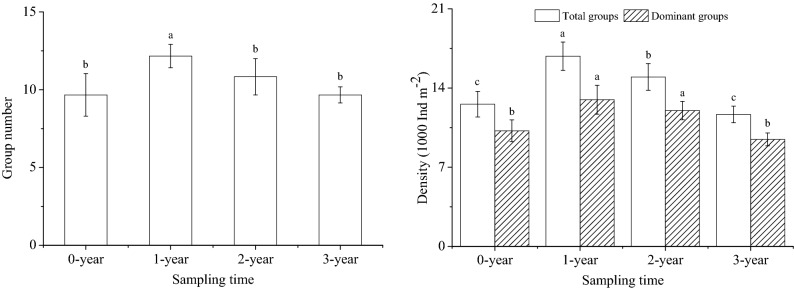
Group number and density of soil meso- and micro-fauna in different sampling times. 0-year, 1-year, 2-year and 3-year represent before planting, planting for one year, two years and three years, respectively. Different lowercase letters indicate significant differences among different sampling times (*P* < 0.05).

In this study, 2968 individuals belonging to 8 classes and 13 groups were identified in the four sampling times. The mean density of soil fauna was 14,005.67 individuals m^-2^, ranging from 11,663.15 individuals m^-2^ in 3-year to 16,815.27 individuals m^-2^ in 1-year (Fig. 1). The communities were dominated by Acariformes, Parasiformes and Collembola, which accounted for 48.86%, 19.50% and 11.32% of the total individual density, respectively (Table 1). The common groups accounted for 19.92% of total individuals, and the rare group (Julida) only represented 0.40% of the communities (Table 1).

**Table 1:** Composition of soil meso- and micro-fauna in different sampling times.

Groups	0-year		1-year		2-year		3-year		Mean	
	Density (Ind m^−2^)	Relative abundance (%)	Density (Ind m^−2^)	Relative abundance (%)	Density (Ind m^−2^)	Relative abundance (%)	Density (Ind m^−2^)	Relative abundance (%)	Density (Ind m^−2^)	Relative abundance (%)
Acariformes	6058.03	48.20***	7784.85	46.30***	7416.84	49.53***	6114.65	52.43***	6843.60	48.86***
Parasiformes	2689.31	21.39***	3283.79	19.53***	2887.47	19.28***	2066.53	17.72***	2731.78	19.50***
Collembola	1472.05	11.71***	1896.67	11.28***	1698.51	11.34***	1273.89	10.92***	1585.28	11.32***
Diptera	481.25	3.83**	962.49	5.73**	764.33	5.10**	566.17	4.85**	693.56	4.95**
Symphyla	481.25	3.83**	537.86	3.20**	368.01	2.46**	452.94	3.88**	460.01	3.29**
Diplura larvae	169.85	1.35**	424.63	2.53**	396.32	2.65**	283.09	2.43**	318.47	2.27**
Coleoptera larvae	283.09	2.25**	339.70	2.02**	311.39	2.08**	283.09	2.43**	304.32	2.17**
Coleoptera adult	254.78	2.03**	283.09	1.68**	368.01	2.46**	198.16	1.70**	276.01	1.97**
Pseudoscorpiones	254.78	2.03**	283.09	1.68**	254.78	1.70**	84.93	0.73*	219.39	1.57**
Scolopendromorpha	113.23	0.90*	311.39	1.85**	198.16	1.32**	113.23	0.97*	184.01	1.31**
Protura	0	0*	481.25	2.86**	0	0*	84.93	0.73*	141.54	1.01**
Pauropoda	311.39	2.48**	113.23	0.67*	226.47	1.51**	113.23	0.97*	191.08	1.37**
Julida	0	0*	113.23	0.67*	84.93	0.57*	28.31	0.24*	56.62	0.40*

*Rare groups, **Common groups, ***Dominant groups.

0-year, 1-year, 2-year and 3-year represent before planting, planting for 1 year, 2 years and 3 years, respectively.

The group number and density of soil fauna varied widely among different sampling times. With the increase of planting years, the group number and density of soil fauna increased first and then decreased. The highest group number and density of soil fauna were found in 1-year (Fig. 1), which was significantly higher than other sampling times (*P* < 0.05). No significant difference was found between 0-year and 3-year (*P* > 0.05). The density of dominant groups showed the same changes with the total density, while there was no significant difference between 1-year and 2-year (*P* > 0.05).

### Diversity characteristics

The diversity characteristics of soil fauna in different sampling times are listed in Table 2. The Shannon-Weiner diversity index (H), Margalef richness index (D) and Density-Group diversity index (DG) increased first and then decreased with the increase of planting years, and the highest values were found in 1-year, with averages of 1.72, 2.43 and 8.05, respectively. The Pielou evenness index (J) decreased with the increase of planting years, and no significant difference was found among the four sampling times (*P* > 0.05). The Simpson dominance index (C) showed an opposite trend to H, and it was significantly lower in 1-year than that in other sampling times (*P* < 0.05).

**Table 2:** Diversity characteristics of soil meso- and micro-fauna in different sampling times.

Sampling time	H	J	C	D	DG
0-year	1.58 ± 0.08b	0.70 ± 0.01a	0.30 ± 0.01b	2.02 ± 0.33c	5.54 ± 1.07b
1-year	1.72 ± 0.08a	0.69 ± 0.02a	0.27 ± 0.02c	2.43 ± 0.15a	8.05 ± 1.25a
2-year	1.61 ± 0.07b	0.68 ± 0.01a	0.30 ± 0.01b	2.19 ± 0.23b	6.92 ± 1.59a
3-year	1.53 ± 0.09b	0.68 ± 0.04a	0.33 ± 0.04a	2.05 ± 0.14c	4.67 ± 0.32b

0-year, 1-year, 2-year and 3-year represent before planting, planting for one year, two years and three years, respectively. H, Shannon-Weiner diversity index; J, Pielou evenness index; C, Simpson dominance index; D, Margalef richness index; DG, Density-Group diversity index. Different lowercase letters in the same column indicate significant differences among different sampling times (*P* < 0.05).

### Soil properties

Different planting years showed significant impact on soil physicochemical properties (Table 3). With the increase of planting years, soil moisture content (SMC) increased first and then decreased, peaking at 1-year, while no significant difference was found among the four sampling times (*P* > 0.05). The pH value increased with the increase of planting years, and 2-year and 3-year were significantly higher than 0-year and 1-year (*P* < 0.05). Contents of SOM, TN, TP and TK increased first and then decreased with the increase of planting years. Contents of TN and TP were significant higher in 1-year than those in other sampling times (*P* < 0.05). Contents of SOM and TK in 1-year and 2-year showed no significant difference (*P* > 0.05), while they were significantly higher than those in 0-year and 3-year (*P* < 0.05). There was no significant difference in C/N among all sampling times (*P* > 0.05).

**Table 3:** Main soil physicochemical properties in different sampling times.

Soil properties	0-year	1-year	2-year	3-year
SMC (%)	33.57 ± 5.07a	38.05 ± 5.73a	36.14 ± 4.34a	33.85 ± 3.32a
pH	4.77 ± 0.14c	4.89 ± 0.14b	5.01 ± 0.12a	5.05 ± 0.09a
SOM (g kg^−1^)	44.88 ± 4.22c	66.57 ± 8.24a	62.45 ± 4.17a	53.29 ± 6.83b
TN (g kg^−1^)	2.23 ± 0.28c	3.14 ± 0.53a	2.83 ± 0.74b	2.41 ± 0.27c
C/N	11.74 ± 0.88a	12.73 ± 3.47a	13.69 ± 4.25a	12.83 ± 0.65a
TP (g kg^−1^)	0.26 ± 0.04c	0.35 ± 0.07a	0.29 ± 0.02b	0.27 ± 0.05c
TK (g kg^−1^)	23.97 ± 1.88b	26.97 ± 4.09a	25.58 ± 2.43a	23.05 ± 2.14b

0-year, 1-year, 2-year and 3-year represent before planting, planting for one year, two years and three years, respectively. SMC, soil moisture content; SOM, soil organic matter; TN, total nitrogen; TP, total phosphorus; TK, total potassium; C/N, ratio of carbon to nitrogen. Different lowercase letters in the same row indicate significant differences among different sampling times (*P* < 0.05).

### Effects of soil properties on soil fauna community

**Figure 2:**
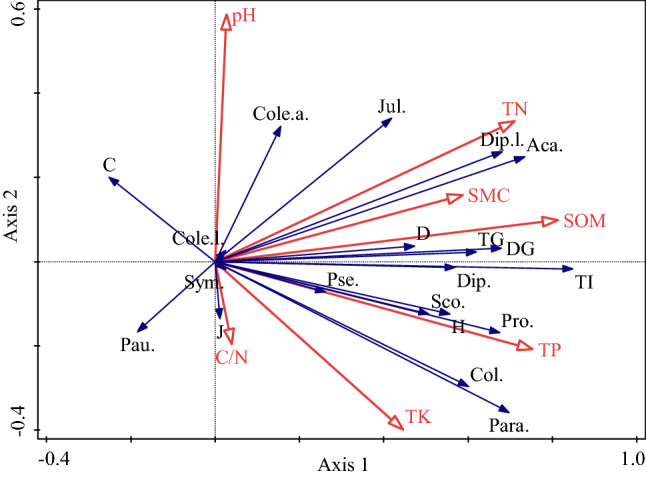
Results of redundancy analysis of soil fauna groups and diversity characteristics in association with soil properties. Hollow arrow points represent soil properties labeled as: soil moisture content (SMC), pH, soil organic matter (SOM), total nitrogen (TN), total phosphorus (TP), total potassium (TK) and ratio of carbon to nitrogen (C/N). Solid arrow points represent groups and diversity characteristics of soil fauna labeled as: Aca., Acariformes; Para., Parasiformes; Col., Collembola; Dip., Diptera; Sym., Symphyla; Dip.l., Diplura larvae; Cole.l., Coleoptera larvae; Cole.a., Coleoptera adult; Pse., Pseudoscorpiones; Sco., Scolopendromorpha; Pro., Protura; Pau., Pauropoda; Jul., Julida; TG, total groups of soil fauna community; TI, total individuals of soil fauna community; H, Shannon–Weiner diversity index; J, Pielou evenness index; C, Simpson dominance index; D, Margalef richness index; DG, Density-Group diversity index.

Redundancy analysis (RDA) showed that the soil fauna explained 68.30% of the total variation in the measured soil properties (Fig. 2). The first canonical axis was mainly determined by SOM, TP, TN and SMC, and explained 65.24% of total variation. The second canonical axis included pH and C/N, and explained 1.81% of total variation. SOM explained the largest variation of soil fauna community variation (43.2%), indicating that SOM was the main environmental factor driving the variation of soil fauna community (Table 4). The explanatory degrees of TP and TN were more than 30%, which could be considered to have a certain impact on the variation of soil fauna community (Table 4). The number of individuals of Acariformes, Parasiformes, Collembola, Scolopendromorpha, Protura, and TI were significantly positively correlated with SOM and TP, while TG, TI, H, D and DG were significantly positively correlated with TN (Fig. 2, Table 5).

**Table 4:** Explanatory degrees of soil fauna community variation of various soil environmental factors by redundancy analysis.

Soil properties	Explains (%)	F value	*P* value
SOM	43.2	16.7	0.002
TP	37.2	13.0	0.004
TN	33.2	11.0	0.002
SMC	22.7	6.5	0.012
TK	13.5	3.4	0.060
pH	0.8	0.2	0.762
C/N	0.5	0.1	0.886

**Table 5:** Person correlation coefficients between soil fauna and soil properties.

Variables	SMC	pH	SOM	TN	TP	TK	C/N
Acariformes	0.503*	0.160	0.646**	0.585**	0.522**	0.188	0.020
Parasiformes	0.391	−0.224	0.563**	0.351	0.594**	0.411*	0.159
Collembola	0.285	−0.117	0.517**	0.319	0.602**	0.337	0.134
Diptera	0.343	0.067	0.565**	0.219	0.400	0.342	0.355
Symphyla	0.113	−0.315	−0.100	0.032	−0.145	0.007	−0.078
Diplura larvae	0.326	0.217	0.498**	0.601**	0.301	0.409*	−0.101
Coleoptera larvae	−0.320	0.165	−0.172	0.203	−0.175	0.401	−0.344
Coleoptera adult	−0.036	0.097	0.135	0.403	0.096	−0.104	−0.271
Pseudoscorpiones	0.151	0.030	0.062	0.161	0.197	0.292	−0.122
Scolopendromorpha	0.365	0.199	0.528**	0.268	0.471*	0.187	0.156
Protura	0.429*	−0.164	0.554**	0.563**	0.652**	0.119	−0.117
Pauropoda	0.043	−0.079	−0.344	−0.267	0.037	0.025	−0.100
Julida	−0.071	0.188	0.268	0.623**	0.029	0.327	−0.311
TG	0.188	0.288	0.554**	0.532**	0.391	0.393	−0.041
TI	0.489*	0.014	0.680**	0.602**	0.639**	0.395	0.025
H	0.133	0.040	0.313	0.482**	0.363	0.497*	−0.210
J	−0.011	−0.443*	−0.345	−0.029	0.056	0.299	−0.280
C	−0.102	0.214	−0.221	−0.293	−0.391	−0.540**	0.097
D	0.067	0.341	0.453*	0.452*	0.270	0.335	−0.062
DG	0.279	0.124	0.464*	0.586**	0.401	0.453*	−0.161

*P < 0.05; **P < 0.01.

## Discussion

### Effects of agroforestry management on soil properties

Previous study showed that bamboo-fungus agroforestry management could change the soil micro-environment^9^. Interplanting edible fungi in moso bamboo forest improved soil quality and soil fertility due to the decomposition of the residue^27^. In our study, contents of SOM and nutrients (TN, TP and TK) reached the maximum in 1 year and subsequently decreased. The increase of SOM and nutrients contents may be explained by different mechanisms as reported earlier. Firstly, the decomposition of mulches (rice chaff and straw) may improve the contents of SOM and nutrients^27^. Secondly, the growth of hypha promoted the formation of soil aggregate structure and increased the porosity of soil^28^. Additionally, the increase of SOM and nutrients contents increased the microbial biomass and soil enzyme activities, and then promoted the decomposition of residues^27^. Finally, microbial residues produced by the iterative cycle of microbial growth, death and turnover were important part of SOM^29^. With the extension of planting years and the decomposition of mulches, the contents of SOM and nutrients gradually decreased and restored to that before planting.

In our study, we also found that the pH value increased with the increase of planting years, indicating that planting *Stropharia rugosoannulata* in moso bamboo forest could effectively reduce soil acidification. Our results disagree with the result of Zhao et al. in moso bamboo forest, who found a decline trend with the increase of mulching years^2^. Researches showed that SOM accumulation could mitigate soil acidification, and the acidity might be neutralized by net mineralization of SOM^30,31^. The introduction of mulches was beneficial to the increase of species and quantity of soil microorganisms, and the increase of biological activity promoted the mineralization of organic nitrogen and the consumption of protons, which increased the pH value^9^. In addition, the decomposition of organic matter increased the organic anions, which was conducive to neutralize acidity^30,32^.

### Link between soil fauna and soil properties

Previous studies revealed that the numbers of groups and individuals of soil fauna were mainly affected by soil environmental factors^33^. Bamboo-fungus agroforestry management significantly increased the numbers of groups and individuals of soil fauna. In this study, the quantity of soil fauna groups and individuals in planting *Stropharia rugosoannulata* stands were higher than in pure moso bamboo forest. The increase of the content of SOM, which provided more food for soil fauna, was conducive to the survival and reproduction of soil meso- and micro-fauna, and increased the soil fauna groups and individuals^23^. This was consistent with the result of Luo et al., who found an increase trend of soil fauna after straw returning to the field^34^. With the increase of planting years, soil fauna groups and individuals first increased and then decreased, which may be related to the decomposition and consumption of organic mulches. The increase of soil fauna groups and individuals, in turn, accelerated the decomposition of mulches^35^, which is not conducive to the activities and reproduction of soil fauna, resulting in the decrease of soil fauna groups and individuals.

The result demonstrated that the diversity indices of soil fauna differed significantly among the four sampling times. The Shannon-Weiner diversity index, Margalef richness index and Density-Group diversity index were significantly higher in 1-year than that before planting, indicating that bamboo-fungus agroforestry management significantly improved the diversity of soil meso- and micro-fauna community. However, the Simpson dominance index was significantly lower in 1-year than other sampling times. The reason for this may be that the cultivation of edible fungi decreased the relative abundances of dominant groups, and increased the relative abundances of common and rare groups (Table 1). The dominant groups of soil meso- and micro-fauna in the test site were Acariformes, Parasiformes and Collembola, accounting for 48.86%, 19.50% and 11.32% respectively and there was no significant difference among four sampling times. The results indicated that bamboo-fungus agroforestry management unchanged the dominant groups of soil fauna in moso bamboo forest.

Previous studies showed that the community structures and compositions of soil fauna communities were associated with soil nutrients and biochemical factors under the influences of factors and their interactions^18,33,36^. In our study, a significant correlation between main soil properties (especially SOM) and indices of soil fauna indicators, such as the individuals of Acariformes, Parasiformes and Collembola, TG, TI, H, D and DG (Table 4, Fig. 2). This illustrated that these soil fauna indices were relatively sensitive to the main soil properties, and could be applied to indicate changes in soil fertility such as SOM^23^. Redundancy analysis (RDA) showed that SOM was the main environmental factor driving the variation of soil fauna community, which explained 43.2% of soil fauna community variation. This result was consistent with Yin et al., who found the soil fauna communities significantly correlated with the content of SOM^33^.

## Conclusion

This study provided an insight into soil meso- and micro-fauna community as affected by planting *Stropharia rugosoannulata* in moso bamboo forest. With the increase of planting years, the group number, density and diversity of soil fauna and major soil nutrients increased first and then decreased, and the highest was found in 1 year after planting, illustrating that bamboo-fungus agroforestry management was beneficial for the diversity and abundance of soil fauna community. Although the individuals of dominant groups increased and their relative abundances decreased, the dominant groups remained unchanged. There was a significant correlation between main soil properties (SOM, TP and TN) and indices of soil fauna indicators. SOM was found to be the main environmental factor driving the variation of soil fauna community.

## Materials and methods

### Study site

The study site is located at Yaowu village (119°75′–119°82′E, 30°60′–30°64′N), Huzhou city, Zhejiang province, China. The region has a subtropical monsoon climate, with an annual average temperature of 15.4 °C, a mean precipitation of 1379 mm and 235 frost-free days. The experimental site is 246 m above sea level with a slope of 17°. The soil is sandy loam, which is defined as Ultisol according to the USDA soil classification system.

### Experimental design

This study was conducted in a pure moso bamboo forest. Three experimental plots (20 m × 20 m) were established as three replications. The diameter at breast height (DBH, 1.3 m) and height of all plants within a plot were measured. Additionally, the age of all plants were estimated expressed by “du”. Bamboo of 1 (I) “du” corresponds to 1–2 years, and consequently, 2 (II) and 3 (III) “du” indicate 3–4 and 5–6 years, respectively^3,4^. The moso bamboo forest was characterized by a stand density of 1576 individuals ha^−1^, a mean height of 13.8 m, an average DBH of 10.9 cm, and a canopy cover of 70%. The age structure was 4:3:3 (I:II:III).

In October 2017, weeds and shrubs in moso bamboo forest were removed, and furrows (0.3 m in width and 0.2 m in depth) along the contour were established. The distance between two adjacent furrows was about 1 m. *Stropharia rugosoannulata* was planted in moso bamboo forests in November 2017 after the application of 7.5 kg m^−2^ of rice chaff in the furrows. The furrows were than covered by straw at 7.5 kg m^−2^ and 5 cm soil. After the edible fungi were harvested, no continuous cultivation was done. In order to maintain a stable bamboo density, bamboo timber cutting was carried out every year.

### Soil sampling and measurement

Soils were sampled annually from 2017 to 2020. Four sampling times were set up, namely (1) before planting as control (October 2017, 0-year); (2) planting for one year (October 2018, 1-year); (3) planting for two years (October 2019, 2-year); and (4) planting for three years (October 2020, 3-year).

Before soil sampling, litters and mulches (rice chaff and straw) were removed from the plot to prevent the contamination of the organic matters to surface soil. Soil samples (0–10 cm) were collected randomly using a soil sampler (5 cm in diameter) at the furrows. Soils from three sample points (planting furrows) in the same plot were mixed as one soil sample. For each plot, three replicated soil samples were sealed separately in three bags, placed in a cooler and transported to laboratory immediately. Soil meso- and micro-fauna community was determined by modified Tullgren techniques^18,37^. The soil meso- and micro-fauna were divided into dominant groups (the number of individuals represented over 10% of the total sample), common groups (the number of individuals represented between 1 and 10% of the total sample) and rare groups (the number of individuals represented less 1% of the total sample).

Similarly, another three soil samples (0–10 cm) were collected near the previous sample points in each plot. Samples were divided into two parts: one part was placed in an aluminum box for the determination of soil moisture content (SMC); the other part was air-dried at room temperature, ground and sieved through 2-mm and 0.15-mm meshes before chemical analysis. The identifiable plant residues, stones and root fragments were removed during sieving.

In the laboratory, the aluminum boxes were oven-dried at 105 °C for the determination of SMC. Soil pH was measured in a 1:2.5 (m/v) soil/water suspension. Soil organic matter (SOM) content was determined by the H_2_SO_4_-K_2_Cr_2_O_7_ wet oxidation method^38^. Total nitrogen (TN) content was measured by the Kjeldahl’s method^39^. Total phosphorus (TP) content was determined using H_2_SO_4_/H_2_O_2_ digestion followed by colorimetric analysis^23^. Total potassium (TK) content was measured using atomic absorption spectrophotometry after H_2_SO_4_/H_2_O_2_ digestion^23^.

### Calculation methods

The following formulas were used to analyze the diversity of soil meso- and micro-fauna community^23,33,40^.

$${\text{H}} = - \mathop \sum \limits_{{{\text{i}} = 1}}^{{\text{S}}} {\text{Pi lnPi}}$$

$${\text{J}} = {\text{H}}/{\text{lnS}}$$

$${\text{C}} = \sum \left( {{\text{Pi}}} \right)^{2}$$

$${\text{D}} = \left( {{\text{S}} - 1} \right)/{\text{lnN}}$$

$${\text{DG}} = \left( {\frac{{\text{g}}}{{\text{G}}}} \right)\mathop \sum \limits_{{{\text{i}} = 1}}^{{\text{g}}} \left( {\frac{{{\text{DiCi}}}}{{{\text{Di}}_{{{\text{max}}}} {\text{C}}}}} \right)$$

where H is the Shannon–Wiener diversity index; J is the Pielou evenness index; C is the Simpson diversity index; D is the Margalef richness index; DG is the Density-Group diversity index; Pi is the relative percentage of the soil meso- and micro-fauna of type “i” in each plot; S is the number of groups; N is the total number of individuals in the soil meso- and micro-fauna; g is the number of groups in each repetition; G is the total number of groups in each sampling time; Ci/C is the ratio of the “i” group in each sampling time; and Di is the density of the “i” group; and Di_max_ is the maximum density.

### Statistical analysis

One-way analysis of variance (ANOVA) and Duncan’s multiple comparisons were used to analyze the significant differences between sampling times. The differences were considered significant at *P* < 0.05. Figures were prepared using the Origin 8.6 software program. Redundancy analysis (RDA) was used to analyze the relative contributions of soil properties to the communities composition of the soil meso- and micro-fauna.

### Statement

The collection of soil samples was permitted by local famers orally. The study complied with local (Zhejiang Province) and national (China) regulations. All the methods in this manuscript were carried out in accordance with relevant guidelines and regulations.

## Data Availability

The datasets used and/or analyzed during the current study are available from the corresponding author on reasonable request.

## References

[1] Jiang ZH (2007). Bamboo and Rattan in the World.

[2] Zhao J, Wang B, Li Q, Yang H, Xu K (2018). Analysis of soil degradation causes in *Phyllostachys edulis* forests with different mulching years. Forests.

[3] Su W, Fan S, Zhao J, Cai C (2019). Effects of various fertilization placements on the fate of urea-^15^N in moso bamboo forests. For. Ecol. Manag..

[4] Zhao J (2019). Ammonia volatilization and nitrogen runoff losses from moso bamboo forests under different fertilization practices. Can. J. For. Res..

[5] Yin J (2019). Abandonment lead to structural degradation and changes in carbon allocation patterns in Moso bamboo forests. For. Ecol. Manag..

[6] Xu QF (2020). Rapid bamboo invasion (expansion) and its effects on biodiversity and soil processes. Glob. Ecol. Conserv..

[7] Prayogo C, Sholehuddin N, Putra EZHS, Rachmawati R (2019). Soil macrofauna diversity and structure under different management of pine-coffee agroforestry system. J. Degrade. Min. Land Manage..

[8] Coleman BR, Martin AR, Thevathasan NV, Gordon AM, Isaac ME (2020). Leaf trait variation and decomposition in short-rotation woody biomass crops under agroforestry management. Agric. Ecosyst. Environ..

[9] Cai CJ, Fan SH, Liu GL, Wang SM, Feng Y (2018). Research and development advance of compound management of bamboo forests. World Bamboo Rattan.

[10] Song Z (2009). Characteristics of Se-enriched mycelia by *Stropharia rugoso-annulata* and its antioxidant activities in vivo. Biol. Trace Elem. Res..

[11] Wang Q, Zhao Y, Feng X, Ibrahim SA, Huang W, Liu Y (2021). Effects of drying on the structural characteristics and antioxidant activities of polysaccharides from *Stropharia rugosoannulata*. J. Food Sci. Technol..

[12] Yan P, Jiang J, Cui W (2004). Characterization of protoplasts prepared from the edible fungus, *Stropharia rugoso-annulata*. World J. Microbiol. Biotechnol..

[13] Frouz J (2018). Effects of soil macro- and mesofauna on litter decomposition and soil organic matter stabilition. Geoderma.

[14] Lin D (2019). Soil fauna promote litter decomposition but do not alter the relationship between leaf economics spectrum and litter decomposability. Soil Biol. Biochem..

[15] Meehan ML (2020). Response of soil fauna to simulated global change factors depends on ambient climate conditions. Pedobiologia.

[16] Tan B (2020). Soil fauna show different degradation patterns of lignin and cellulose along an elevational gradient. Appl. Soil Ecol..

[17] John K, Zaitsev AS, Wolters V (2021). Soil fauna groups respond differentially to changes in crop rotation cycles in rice production systems. Pedobiologia.

[18] Qin Z (2019). Changes in the soil meso- and micro-fauna community under the impacts of exotic *Ambrosia artemisiifolia*. Ecol. Res..

[19] Chauvat M, Titsch D, Zaytesev AS, Wolters V (2011). Changes in soil faunal assemblages during conversion from pure to mixed forest stands. For. Ecol. Manag..

[20] Yan S (2012). A soil fauna index for assessing soil quality. Soil Biol. Biochem..

[21] Reeve JR (2010). Effects of soil type and farm management on soil ecological functional genes and microbial activities. ISME J..

[22] Lavelle P, Bignell D, Lepage M (1997). Soil function in a changing world: The role of invertebrate engineers. Eur. J. Soil Biol..

[23] Zhu X, Zhu B (2015). Diversity and abundance of soil fauna as influenced by long-term fertilization in cropland of purple soil, China. Soil Till. Res..

[24] Zhang L, Wang G, Cao F (2015). The effect of ginkgo agroforestry patterns on soil fauna diversity. J. Nanjing For. Univ..

[25] Liu P (2018). Impact of straw returning on cropland soil mesofauna community in the western part of black soil area. Chin. J. Ecol..

[26] Liu, M. Study on the model of interplanting edible fungi under bamboo (*Phyllostachys edulis*) forest and comprehensive benefit comparative. Master’s Thesis, Chinese Academy of Forestry (2021) (**in Chinese**).

[27] Wang B, Shen Q, Zhu W, Shen X, Li Q (2016). Effects of interplanting *Dictyophora echinovolvata* on physicochemical properties, phospholipid fatty acids characters and enzyme activities in soil of *Phyllostachy heterocycla *cv.* pubescen*s. For. Environ. Sci..

[28] Ying GH (2014). Effect of cultivation of *Dictyophora echinovolvata* on shoot yield and soil under *Phyllostachy heterocycla *cv.* pubescens* stand. J. Zhejiang For. Sci. Technol..

[29] Sokol NW (2022). Life and death in the soil microbiome: How ecological processes influence biogeochemistry. Nat. Rev. Microbiol..

[30] Fujii K, Hayakawa C, Inagaki Y, Kosaki T (2020). Effects of land use change on turnover and storage of soil organic matter in a tropical forest. Plant Soil.

[31] Fujii K, Toma T (2021). Comparison of soil acidification rates under different land uses in Indonesia. Plant Soil.

[32] Poss R, Smith CJ, Dunin FX, Angus JF (1995). Rate of soil acidification under wheat in a semi-arid environment. Plant Soil.

[33] Yin X (2018). Distribution and diversity partterns of soil fauna in different salinization habitats of Songnen Grasslands, China. Appl. Soil Ecol..

[34] Luo ML (2020). Effects of different rice straw returning quantities on soil fauna community structure. J. Zhejiang A&F Univ..

[35] Peng CY (2019). Community structure characteristics of medium- and small-sized soil faunas in typical artificial plantation in the upper reaches of Yangtze River. J. Zhejiang Univ..

[36] Carmen MU, Edmond RZ, Michelle MW (2013). Nematode indicators as integrative measures of soil condition in organic cropping systems. Soil Biol. Biochem..

[37] Kamau S, Karanja NK, Ayuke FO, Lehmann J (2019). Short-term influence of biochar and fertilizer-biochar blends on soil nutrients, fauna and maize growth. Biol. Fertil. Soils.

[38] Fu X, Shao M, Wei X, Horton R (2010). Soil organic carbon and total nitrogen as affected by vegetation types in Northern Loess Plateau of China. Geoderma.

[39] Guan F, Tang X, Fan S, Zhao J, Peng C (2015). Changes in soil carbon and nitrogen stocks followed the conversion from secondary forest to Chinese fir and Moso bamboo plantations. Catena.

[40] Liu Y (2019). Higher soil fauna abundance accelerates litter carbon release across an alpine forest-tundra ecotone. Sci. Rep..

